# *In vitro* and *in vivo* characterization of Bisphosphocin Nu-3—a novel broad-spectrum antimicrobial compound with high potency against resistant pathogens

**DOI:** 10.1128/aac.00948-25

**Published:** 2025-10-21

**Authors:** Kelvin Cooper, Kanneganti Murthy, Thomas Balzer, Steve Parkinson, Randy Sleet, Paul DiTullio

**Affiliations:** 1 Lakewood-Amedex Biotherapeutics Inc., University Park, Florida, USA; University of Fribourg, Fribourg, Switzerland

**Keywords:** antimicrobial resistance, diabetic foot ulcer infections, topical, novel class, diabetes, Gram-positive bacteria, Gram-negative bacteria, multidrug resistance

## Abstract

Lakewood-Amedex Biotherapeutics Inc. has developed a new class of antimicrobials called the Bisphosphocin class, with Nu-3 as a leading candidate, which is currently in clinical development for treating mildly infected diabetic foot ulcers (iDFUs). Nu-3 acts through a novel mechanism of action that destabilizes bacterial membranes within minutes, which significantly reduces the potential for resistance development. It demonstrates broad-spectrum activity against Gram-positive, Gram-negative, and multidrug-resistant bacteria, including recent clinical isolates from infected wounds. Nu-3 is especially effective in acidic environments and can be formulated for topical application at high concentrations and low pH, that has been shown to be beneficial for wound healing, making it well-suited for treating infections in diabetic foot ulcers. Its rapid, bactericidal action has been confirmed against pathogens, such as *Staphylococcus aureus*, *Escherichia coli*, *Pseudomonas aeruginosa, Klebsiella pneumoniae, Propionibacterium acnes, Proteus mirabilis, Acinetobacter baumannii,* and *Staphylococcus saprophyticus*, with complete bacterial eradication occurring within minutes. Resistance testing over 21 days showed a very low probability of resistance development. *In vivo* testing in murine dermal methicillin-resistant *Staphylococcus aureus* (MRSA) infection models demonstrated robust efficacy for Nu-3 given as a solution- or gel-formulation in both single and multidose protocols. Given its efficacy, broad-spectrum activity, and topical formulation, Nu-3 has the potential to be an important therapeutic intervention for the treatment of mild infections in diabetic foot ulcers—a serious complication of diabetes associated with high amputation risk.

## INTRODUCTION

Antimicrobial resistance (AMR) continues to pose a significant global public health threat, ranking among the top 10 challenges faced by humanity. A Lancet publication in 2022 on the global burden of AMR, based on data from 2019, reported that 4.95 million deaths were associated with antimicrobial resistance, with 1.27 million deaths directly attributable to AMR (1). The CDC reported that in the US alone, more than 2.8 million AMR infections occur each year, with more than 35,000 deaths as a result (2). Beyond its devastating impact on human lives, AMR may also jeopardize the global economy, with implications for international trade, health care expenditures, and overall productivity. If left unaddressed, by 2050, the economic toll of AMR could reach a staggering US$3.8 trillion in GDP loss (3). Importantly, treatment of infections caused by the six leading multidrug-resistant pathogens cost more than $4.6B in 2021 in the US (4).

As a result of drug resistance, antibiotics and antifungal medicines become ineffective, and infections caused by resistant pathogens become more difficult or impossible to treat, increasing the risk of disease spread, severe illness, disability, and death. This situation is predominantly linked to the misuse and/or overuse of antibiotics to treat, prevent, or control infections in humans, animals, and plants (entering the food chain). In addition, bacteria are able to form biofilms and an acidic micro-environment that further limits or prevents treatment with conventional antibiotics (5). Unfortunately, the discovery and launch of antimicrobial drugs has slowed to a trickle while resistant organisms have emerged for most classes of antibiotics. It is clear that new classes of AMR drugs are urgently needed (2).

Diabetic foot ulcers (DFU) represent a complex and difficult-to-treat complication of diabetes mellitus and include three major pathologic conditions that appear as the main contributors to the development of DFUs: ischemia, neuropathy, and infection. Infection control is accepted to be a key success factor in facilitating healing and preventing disease progression to a more severe form of infection, which eventually may lead to amputation (6), and in extreme cases, when left untreated, can even lead to death. Diabetic foot ulcer infections are frequently polymicrobial, comprising both Gram-positive and Gram-negative bacteria, including multidrug-resistant bacteria. The most common Gram-positive bacteria found are *Streptococcus* spp., *Staphylococcus* spp., and *Enterococcus* spp.*,* and the most common Gram-negative bacteria found are *Escherichia coli, Klebsiella pneumoniae, Morganella morganii, and Proteus mirabilis,* with other Gram-negative bacteria, such as *Citrobacter, Enterobacter cloacae,* and *Pseudomonas aeruginosa,* being found less frequently (7, 8).

Lakewood-Amedex Biotherapeutics Inc. has discovered a new class of antimicrobials called the Bisphosphocin class of antimicrobial agents (9, 10). Unlike traditional antibiotics, Bisphosphocin compounds possess a novel mechanism of action that involves destabilizing bacterial membranes within minutes, frequently in under a minute, leading to the potential for a reduced probability of resistance development, which occurs from prolonged exposure to antibiotics, resulting in selection of resistant bacteria surviving while more susceptible, weaker bacteria are eliminated. This unique mechanism makes the Bisphosphocin class highly effective, and additional profiling of Nu-3 has revealed an extremely broad spectrum of antimicrobial activity encompassing Gram-positive, Gram-negative, and multidrug-resistant bacteria, a rapid cidal action, and a very low potential for generating resistance development. In addition, Nu-3 demonstrates improved microbiological activity at low pH values and can be formulated for topical application in high concentrations and at low pH. In summary, the combination of characteristics of Nu-3 makes it a promising candidate for the treatment of infections in diabetic foot ulcers, and as a result, the compound has advanced into clinical development. The *in vitro* profile of Nu-3, including activity against recent clinical isolates from infected wounds and the *in vivo* efficacy as a topical agent in the treatment of murine methicillin-resistant *Staphylococcus aureus* (MRSA) dermal infections, is discussed.

## MATERIALS AND METHODS

### Organisms

The bacterial strains used were clinical isolates obtained from either the American Type Culture Collection, from the Micromyx collection, or from geographically separate medical centers across the United States.

### Antibacterial agent Nu-3

Nu-3 was synthesized at Aragen Life Sciences in Hyderabad, India, under cGMP guidelines (11). The reference drugs meropenem and levofloxacin were obtained from Micromyx; ciprofloxacin was obtained from Goldbio; oxacillin was obtained from United States Pharmacopeia; vancomycin was obtained from Sigma-Aldrich; and Soframycin was obtained from Sanofi India Ltd.

### Chemical structure of Nu-3

**Fig 1:**
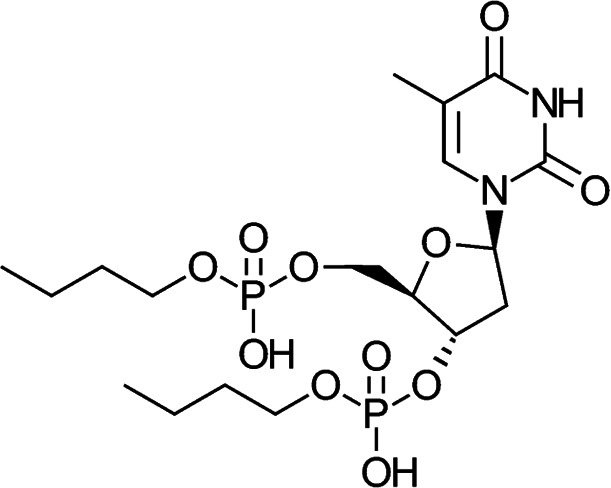
Chemical structure of Nu-3.

1-[(2*R*,4*S*,5*R*)-4-(hydroxybutoxyphosphoryloxy)-5-[(hydroxybutoxyphosphoryloxy)methyl]tetrahydro-2-furyl]-5-methyl-2,4(1*H*,3*H*)-pyrimidinedione (see Fig. 1).

### Formulations

Stock solutions were prepared by dissolving Nu-3 disodium salt in water, and the pH was adjusted with either HCl solution or NaOH solution to achieve the targeted pH. The gel formulations were manufactured at Pace Life Sciences in Michigan, USA. Appropriate quantities of Nu-3 disodium salt were dissolved in sterile water, and the pH was adjusted to 1.5 with 10% hydrochloric acid. The Nu-3 solutions were heated to 60°C and added to a heated, molten mixture of Crodacol CS50 and Cetomacrogol 1000, also at 60°C. The resulting mixture was homogenized to a gel consistency and allowed to cool before being stored as a sterile gel in sterile containers, generating 2%, 5%, and 10% gels (20, 50, and 100 mg/mL, respectively).

### Test media

Cation-adjusted Mueller-Hinton broth (CAMHB; BD) was used for minimum inhibitory concentration (MIC) testing of aerobic organisms. For *Streptococcus* and *Corynebacterium* isolates, this medium was supplemented with 3% laked horse blood (LHB; Hemostat; Dixon, CA). For *Haemophilus influenzae*, *Haemophilus* Test medium (HTM) was used. HTM was made by supplementing CAMHB with 15 µg/mL nicotinamide adenine dinucleotide (NAD; Sigma; St. Louis, MO; Lot. No. W58778), 15 µg/mL hematin porcine (Sigma), and 5 g/L of yeast extract (Sigma).

### Susceptibility tests

MIC values were determined using a broth microdilution procedure described by the Clinical and Laboratory Standards Institute (CLSI) (12). Ninety-six-well MIC plates were prepared using automated liquid handlers for serial dilutions and liquid transfers. Test agents were added to the plates at 10 times the highest final concentration to be tested. Serial twofold dilutions were made across the rows, and daughter plates were then created containing 100 µL of 2× drug solution. A standardized inoculum of each test organism was prepared per CLSI methods to equal a 0.5 McFarland standard into 0.9% sterile saline, followed by an additional 1:10 dilution in saline. The plates were then inoculated with 10 µL of the inoculum suspension, resulting in a final concentration of approximately 5 × 10^5^ colony-forming units (CFU)/mL per well for bacteria. Following the inoculation, the plates were incubated aerobically at 35°C, and after 30 min, the plates were removed from the incubator, and the Multidrop 384 was used to add an additional 90 µL of 2.1× of appropriate test media to reach the final assay volume of ~0.2 mL.

In addition, a standard MIC was conducted for QC organisms and comparator drugs. Both sets of plates were incubated at 35°C for approximately 16 to 20 h (aerobes), 20 to 24 h (*Streptococcus* and *Haemophilus*), and 24–48 h (*Corynebacterium*). Following incubation, the microplates were removed from the incubator and viewed from the bottom using a plate viewer. The MIC was read and recorded as the lowest concentration of drug that inhibited visible growth of the organism. For full details of the MIC tests, see the Supplementary materials.

### Time-kill studies

Time-kill assays were performed similarly to MIC assays using two different pH/time protocols to determine how pH impacts Nu-3 activity and to inform the selection of the formulation properties for Nu-3. In the first protocol, bacteria were cultured overnight in bovine heart infusion (BHI) media and then diluted to a concentration of 1.0 × 10^6^ CFU/mL in saline. Nu-3 stock solutions (10%, pH 1.5) were diluted in water to achieve a concentration that was double the intended final concentration. The concentration range was selected to span the MIC values for Nu-3 against the bacteria chosen and to also inform the selection of the formulation properties for Nu-3. Both the bacterial and drug solutions were prepared immediately before use and kept at room temperature. Equal volumes of the bacterial and drug solutions were mixed in an Eppendorf tube, resulting in a final concentration of 5 × 10^5^ CFU/mL of bacteria, which were then incubated at room temperature. At designated time points (1 and 10 min), 5 µL of the reaction mixture was plated onto BHI agar plates and incubated overnight at 37°C. The concentration at which no bacterial growth was observed is considered to represent 100% killing.

In the second protocol, the time-kill assay was performed at a 10% concentration of test compound Nu-3 at pH 3.5. 25 µL of bacterial culture was added to 225 µL of test solution at 10% in Eppendorf tubes and incubated at room temperature. Bacterial growth was quantified at 1, 5, 10, and 20 min at room temperature. At each time point, 5 µL of culture was withdrawn and diluted 10-fold with 45 µL of saline, and 50 µL of diluted and undiluted samples were plated on pre-incubated sterile Luria Bertani Agar plates and incubated overnight at 37°C, along with a vehicle control (pH 3.5 adjusted saline with HCl). After incubation, the plates were checked for colonies, counted, and recorded. The experiment was done in duplicate.

### Resistance development studies

The *in vitro* emergence of resistance assay was performed by serial passaging cultures of *S. aureus* (ATCC-43300, MRSA) and *E. coli* (ATCC-25922) that had been exposed to sub-MIC levels of the test compounds for 21 cycles. Ciprofloxacin was used as the positive control. The initial determination of the test compound’s MIC was conducted in 5 mL polycarbonate culture tubes containing 1 mL of LB broth, utilizing the microbroth dilution technique. For the subsequent passages, the bacterial inoculum was sourced from the previous passage tubes that demonstrated visual growth at sub-MIC concentrations. The serial passage experiment was extended to 21 cycles. Daily, 1 µL culture was transferred to 1 mL fresh medium containing the appropriate drugs. The concentration ranges of the test compound were modified throughout the study as the MIC values increased. The MIC values of each compound were determined after each cycle (daily), and each assay was conducted in duplicate in Luria broth (LB) using the microbroth dilution method described by the CLSI. To observe small changes in the MIC values, smaller increments in antimicrobial concentrations were employed compared to those generally utilized for standard MIC measurements (for example, Nu-3 serial dilutions used in the assay were 15, 12.5, 10, 7.5, and 5 mg/mL). Bacterial samples were incubated at 37°C for 18 to 24 h with gentle shaking during the MIC assays. The MIC was defined as the lowest concentration of the drug that resulted in no visible growth in the broth.

### Single and multiple dose murine dermal infection models using methicillin-resistant *S. aureus* (USA300 and ATCC 43300)

Female SKH-1 hairless mice (euthymic and immunocompetent, Charles River Breeding Laboratories), aged 6–8 weeks old, were used in all tests. The mice were anesthetized using an isoflurane induction chamber, and the dorsal area underwent 7–10 applications and removals of surgical tape to remove the outermost epidermal layer of skin cells (tape stripping). The infecting inoculum of methicillin-resistant *S. aureus* (USA300 or ATCC 43300) was prepared from an overnight culture to yield an undiluted bacterial concentration of approximately 10^9^ CFU. The cultures were then diluted to provide a challenge inoculum of 6.0 log_10_ CFU per mouse. Immediately following tape stripping, each animal received a topical application of the inoculum spread over the abraded area. The challenge was allowed to dry slightly before the mice were brought out of anesthesia. Application of the bacterial challenge constituted time zero for each study. Four hours following the challenge, mice were treated with the test article or vehicle.

At the scheduled times following treatment, the mice were euthanized, and a section of excised skin approximately 0.5″ × 0.5″ was aseptically removed from the infected/treated area and transferred to vials with 2.0 mL of sterile water and weighed. Tissues were allowed to set at room temperature for 10 min. Tissues were homogenized using a bead beater. The homogenate was serially diluted from neat to 10^−7^ in PBS and plated in duplicate 5 µL spots onto trypticase soy agar plates supplemented with 5% sheep blood cells. The undiluted (neat) homogenate was plated in a 100 µL volume for each sample. Plates were incubated overnight at 37°C in ambient atmosphere. CFUs were tabulated for each treatment per gram of tissue. All experiments included a control group of animals that were infected on the tape-stripped skin but received no treatments and were considered the infection control animals. In addition, for experiments using Nu-3 solution as the treatment, an additional control group was included of animals that were infected on the tape-stripped skin and were treated with pH-adjusted saline to match the pH of the solution being used as the active treatment—the saline control group. Similarly, for experiments using Nu-3 gel as the treatment, an additional control group was included of animals that were infected on the tape-stripped skin and were treated with pH-adjusted placebo gel to match the pH of the solution—the vehicle control group. All data analysis of the studies was performed using a one-way analysis of variance (ANOVA) at a 95% confidence level.

The single-dose studies on 2% and 10% Nu-3 solutions and the 10% Nu-3 gel were conducted by TransPharm Preclinical Solutions in Jackson, Michigan, with four animals per group and were approved by the TransPharm Institutional Animal Care and Use Committee and conducted under veterinary license number 6901009609.

The single-dose study comparing 10% Nu-3 solution and gel with 1% Soframycin and the multidose study on Nu-3 gel, 5% and 10%, both once a day (QD) and twice a day (BID), with six animals per group, were conducted at TheraIndx LifeSciences Pvt. Ltd. in Bangalore, India. All studies were conducted in accordance with the ethical practices laid down in the CPCSEA guidelines for animal care and use (13), and each study was approved by the Institutional Animals Ethics Committee (IAEC) of the test facility (IAEC approval number—IAEC/22/2022/256) and conducted under the CPCSEA license number 1852/PO/Rc/S/16/CPCSEA.

## RESULTS

The Bisphosphocin class has broad-spectrum activity against all of the most common bacteria found in diabetic foot ulcer infections, such as *Streptococcus* spp., *Staphylococcus* spp., *Enterococcus* spp.*, E. coli, K. pneumoniae, M. morganii, P. mirabilis, Citrobacter, E. cloacae,* and *P. aeruginosa,* and the purpose of the *in vitro* screening was to assess Nu-3 against these bacteria, including resistant organisms with different resistance phenotypes and confirm Nu-3 activity against more recent clinical isolates. Nu-3 was tested as both a solution and as a gel formulation for its ability to treat murine skin infections caused by MRSA to confirm activity as a topical agent.

### *In vitro* activity

#### MIC profiling

For Gram-positive bacteria, Nu-3 at concentrations of 0.78 to 25 mg/mL inhibited the growth of several strains of *Staphylococcus* spp. isolates, *Enterococcus* spp. isolates, *Streptococcus* spp. isolates, and the *Corynebacterium striatum* isolate tested (see Table 1). For Gram-negative bacteria, Nu-3 at concentrations of 0.78 to 6.25 mg/mL inhibited the growth of several strains of *E. coli, K. pneumoniae*, *P. aeruginosa*, *Acinetobacter* spp., *Citrobacter freundii, Serratia marcescens*, *P. mirabilis*, and *M. morganii* (see Table 2). Nu-3 is less potent than the standard antibiotics meropenem and levofloxacin for all susceptible strains but retains identical activity against the resistant strains.

**TABLE 1:** *In vitro* Nu-3 activity against Gram-positive isolates, including resistant organisms and recent clinical isolates, with a range of resistance profiles using the microtiter broth dilution method^*a*^

Organism	Isolate	Resistance type	MIC (mg/mL)				
			Nu3	MEM	LVX	Oxacillin	Vancomycin
*Staphylococcus aureus*	ATCC 29213	MSSA	25	0.00012	0.00025	NT	NT
*Staphylococcus aureus*	ATCC 33591	MRSA	12.5	0.0001	0.00025	NT	NT
*Staphylococcus epidermidis*	ATCC 12228	VSE	3.125	0.00012	0.00025	NT	NT
*Staphylococcus haemolyticus*	ATCC 29970	Susceptible	6.25	0.00025	0.0005	NT	NT
*Staphylococcus saprophyticus*	ATCC 49907	Susceptible	6.25	0.00025	0.0005	NT	NT
*Enterococcus faecalis*	ATCC 29212	VSE	6.25	0.002	0.001	NT	NT
*Enterococcus faecalis*	MMX 3353	*vanB*; VRE	12.5	0.002	0.001	NT	NT
*Enterococcus faecium*	ATCC 35667	VSE	6.25	>0.004	0.008	NT	NT
*Enterococcus faecium*	MMX 3355	*vanA*; VRE	6.25	>0.004	>0.008	NT	NT
*Streptococcus agalactiae*	ATCC 13813	Susceptible	1.56	0.000016	0.0005	NT	NT
*Streptococcus pneumoniae*	ATCC 49619	PISP	1.56	0.00006	0.001	NT	NT
*Streptococcus pneumoniae*	MMX 8137	PRSP	0.78	0.001	0.001	NT	NT
*Streptococcus pyogenes*	ATCC 49399	Susceptible	0.78	0.000008	0.001	NT	NT
*Streptococcus dysgalactiae*	ATCC 6644	Susceptible	1.56	0.000016	0.0005	NT	NT
*Corynebacterium striatum*	ATCC 6940	Susceptible	1.56	0.00012	0.001	NT	NT
Recent clinical isolates							
*Enterococcus faecalis*	1231079^*b*^	VSE	12.5	NT	NT	0.016	0.001
*Enterococcus faecalis*	1231150	VSE	6.25	NT	NT	>0.016	0.001
*Enterococcus faecalis*	1244757	VSE	6.25	NT	NT	>0.016	0.001
*Enterococcus faecalis*	1244798	VSE	6.25	NT	NT	0.016	0.001
*Enterococcus faecalis*	1235228	VRE	6.25	NT	NT	>0.016	>0.032
*Enterococcus faecalis*	1265416	VRE	12.5	NT	NT	>0.016	>0.032
*Staphylococcus aureus*	1230647	MSSA	6.25	NT	NT	0.025	0.0005
*Staphylococcus aureus*	1231136	MSSA	6.25	NT	NT	0.00025	0.0005
*Staphylococcus aureus*	1234299	MSSA	6.25	NT	NT	0.00025	0.001
*Staphylococcus aureus*	1236804	MSSA	6.25	NT	NT	0.001	0.001
*Staphylococcus aureus*	1244470	MSSA	6.25	NT	NT	0.01	0.0005
*Staphylococcus aureus*	1249032	MSSA	6.25	NT	NT	0.00012	0.0005
*Staphylococcus aureus*	1231131	MRSA	6.25	NT	NT	>0.016	0.00025
*Staphylococcus aureus*	1232605	MRSA	6.25	NT	NT	>0.016	0.0005
*Staphylococcus aureus*	1233808	MRSA	12.5	NT	NT	>0.016	0.0005
*Staphylococcus aureus*	1244473	MRSA	6.25	NT	NT	>0.016	0.001
*Staphylococcus aureus*	1244794	MRSA	6.25	NT	NT	>0.016	0.0005
*Staphylococcus aureus*	1249449	MRSA	3.125	NT	NT	>0.016	0.0005
*Staphylococcus aureus*	1250232	MRSA	6.25	NT	NT	>0.016	0.0005
*Staphylococcus aureus*	1256700	MRSA	6.25	NT	NT	>0.016	0.001
*Staphylococcus aureus*	1265381	MRSA	12.5	NT	NT	>0.016	0.0005
*Staphylococcus aureus*	1270046	MRSA	6.25	NT	NT	>0.0016	0.001

^*a*^ MEM, meropenem; LVX, levofloxacin; MSSA, methicillin-susceptible *S. aureus*; MRSA, methicillin-resistant *S. aureus*; VSE, vancomycin-susceptible *Enterococcus*; *vanB*, resistant to vancomycin but susceptible to teicoplanin; VRE, vancomycin-resistant *Enterococcus*; *vanA*, resistant to vancomycin and teicoplanin; PISP, penicillin-intermediate resistant *S. pneumoniae*; PRSP, penicillin-resistant *S. pneumoniae*; NT, not tested.

^*b*^ Recent clinical isolates from wounds, including infected diabetic foot ulcers (see Supplemental material for full details).

**TABLE 2:** *In vitro* Nu-3 activity against Gram-negative isolates, including resistant organisms, with a range of resistance profiles using the microtiter broth dilution method^*a*^

Organism	Isolate	Resistance type	MIC (mg/mL)	MIC (mg/mL)	
			Nu-3	MEM	LVX
*Escherichia coli*	ATCC 25922	QC	6.25	0.0003	0.00003
*Escherichia coli*	MMX 8870	ESBL	6.25	0.00006	>0.008
*Enterobacter cloacae*	ATCC BAA-2468	NDM-1	3.125	>0.004	>0.008
*Klebsiella pneumoniae*	ATCC 43816	Susceptible	3.125	0.00006	0.00006
*Klebsiella pneumoniae*	ATCC BAA-2146	NDM-1	3.125	>0.004	>0.008
*Pseudomonas aeruginosa*	ATCC 27853	Susceptible	1.56	0.005	0.001
*Acinetobacter baumannii*	ATCC 19606	Susceptible	3.125	0.001	0.0005
*Acinetobacter radioresistens*	MMX 8551	Susceptible	3.125	0.00025	0.0005
*Morganella morganii*	CDC 0519	Susceptible	6.25	>0.004	0.00012
*Proteus mirabilis*	CDC 0155	Susceptible	6.25	>0.004	>0.008
*Citrobacter freundii*	CDC 0157	Susceptible	3.125	>0.004	>0.008
*Serratia marcescens*	CDC 0520	Susceptible	3.125	>0.004	>0.008
*Haemophilus influenzae*	ATCC 49247	Susceptible	0.78	0.00012	0.00003

^*a*^ MEM, meropenem; LVX, levofloxacin; QC, quality control; ESBL, extended beta-lactamases; NDM-1, New Delhi metallo-beta-lactamases.

In preparation for clinical testing, Nu-3 was tested against a series of recent clinical strains isolated from wounds and diabetic foot infections during 2022, which include six strains of *Enterococcus faecalis*, four vancomycin-sensitive strains, and two vancomycin-resistant strains, plus 16 strains of *S. aureus*, 11 methicillin-sensitive strains, and five methicillin-resistant strains (see Table S1 for full details of the clinical isolate origins). Nu-3 showed similar activity to all strains irrespective of their resistance profile with MICs ranging from 3.125 to 25 mg/mL (see Table 1).

#### Time-kill profiling

**Fig 2:**
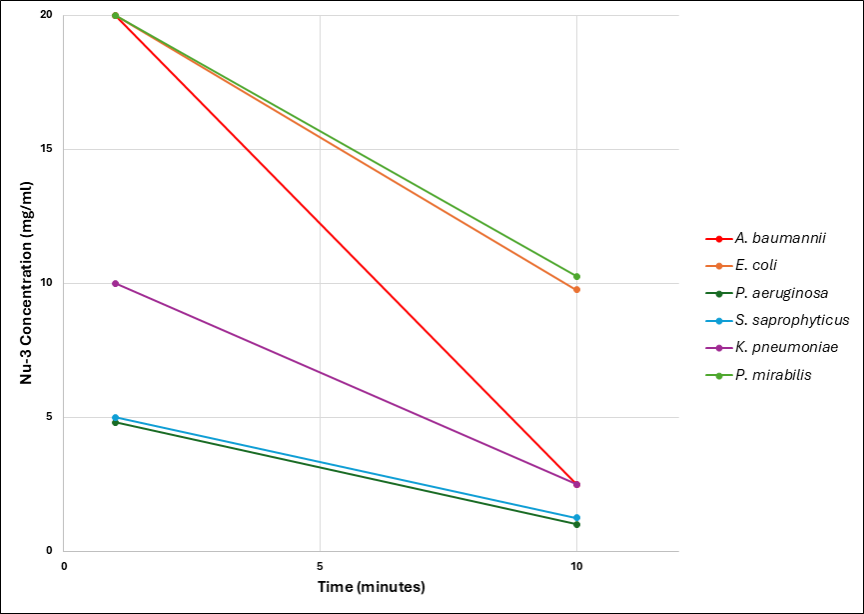
Nu-3 concentrations in mg/mL required to demonstrate complete kill of six strains of bacteria at 1 and 10 min.

The rapid cidal action of Nu-3 is considered an attractive property for the treatment of infections, which would result in fast eradication of infections in infected diabetic foot ulcer (iDFU). Nu-3 was profiled in a concentration-dependent time kill assay against a range of bacteria, including *A. baumannii*, *E. coli*, *P. aeruginosa*, *S. saprophyticus*, *K. pneumoniae,* and *P. mirabilis,* that are frequently found in diabetic foot ulcer infections. Nu-3 was tested at 1 and 10 min to identify the lowest concentration that killed all bacteria. These experiments were conducted at pH 1.5, which is the pH of the gel formulation expected to be used in the forthcoming clinical trial, and the results are represented in Fig. 2 (Table S2). Nu-3 showed a complete kill at 10 min for all the species tested at a maximum of 10 mg/mL.

**Fig 3:**
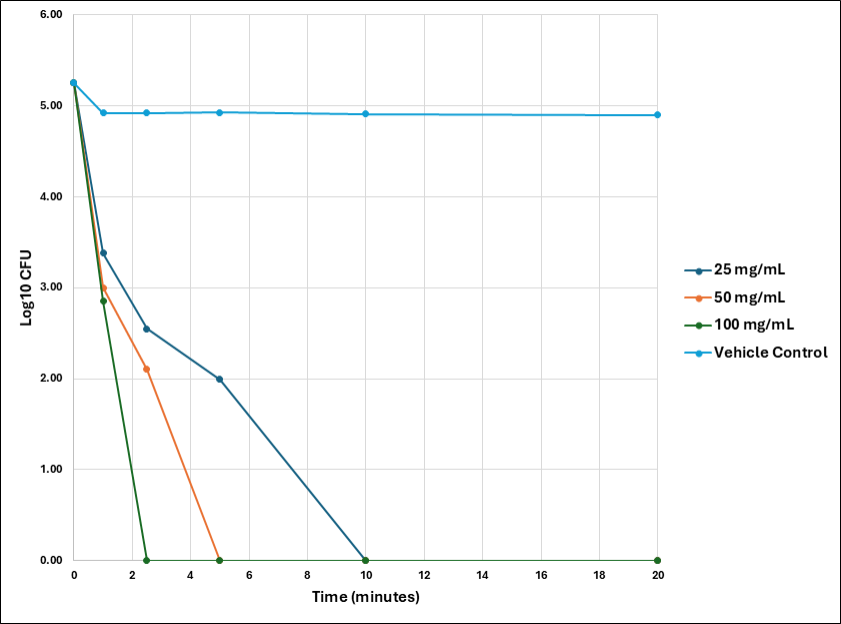
Time kill reduction of *E. coli* (ATCC-25922) CFU vs time for a range of Nu-3 concentrations at pH 3.5.

In addition, Nu-3 was tested against *E. coli* (ATCC 25922) and two strains of *S. aureus* (ATCC 43300 and USA-300) at a higher pH value of 3.5 and at a range of concentrations from 25 to 100 mg/mL commensurate with the expected concentrations of Nu-3 in the gel formulation being used in the forthcoming clinical trial. As shown in Fig. 3, at pH 3.5, Nu-3 showed a time-dependent reduction in both MRSA strains (ATCC 43300 and USA 300) across the time points from 1, 5, 10, and 20 min. In a similar fashion, Nu-3 showed a concentration-dependent inhibitory effect on *E. coli growth* (ATCC 25922) when compared to vehicle control (pH 3.5 adjusted with 1 M citric acid in saline) at the 1 min time point (Table S3).

#### Resistance profiling

**Fig 4:**
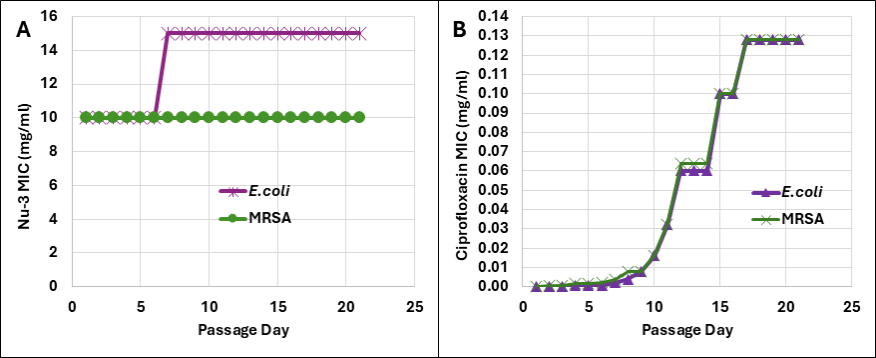
Resistance development assessment assays against *S. aureus* and *E. coli* using a 21-day serial passage experiment as described in the Materials and Methods. (**A**) Nu-3 demonstrates very little change in MIC over the 21-day period, whereas (**B**) ciprofloxacin shows a marked increase in MICs.

Finally, Nu-3 was tested for resistance emergence using serial passage assay against the bacteria *E. coli* (ATCC 25922) and *S. aureus* (ATCC 43300, MRSA) and using ciprofloxacin as the active control. The antimicrobial activity of Nu-3 and ciprofloxacin was evaluated through the measurement of their MICs. The initial MIC values of Nu-3 for *E. coli* and *S. aureus* were both 10 mg/mL, while the corresponding values for ciprofloxacin were 0.00005 and 0.0002 mg/mL, respectively. Following 21 serial passages, the MICs of ciprofloxacin increased to >0.12 mg/mL, representing an increase of more than 2,000-fold for *E. coli* and >600-fold for *S. aureus*. In contrast, the MIC of Nu-3 showed only a slight increase for *E. coli* after a few passages but remained unchanged for *S. aureus* (see Table 3; Fig. 4).

**TABLE 3:** MIC values for Nu-3 and ciprofloxacin in a 21-day serial passage experiment

	MIC			
Day/passage	Nu-3 (mg/mL)		Ciprofloxacin (mg/mL)	
	*E. coli*	*S. aureus*	*E. coli*	*S. aureus*
1	10	10	0.00005	0.0002
2	10	10	0.0001	0.0004
3	10	10	0.0002	0.0008
4	10	10	0.0004	0.0016
5	10	10	0.0004	0.0016
6	10	10	0.0008	0.002
7	15	10	0.002	0.004
8	15	10	0.004	0.008
9	15	10	0.008	0.008
10	15	10	0.016	0.016
11	15	10	0.032	0.032
12	15	10	0.06	0.064
13	15	10	0.06	0.064
14	15	10	0.06	0.064
15	15	10	0.095	0.1
16	15	10	0.095	0.1
17	15	10	0.12	0.128
18	15	10	0.12	0.128
19	15	10	0.12	0.128
20	15	10	0.12	0.128
21	15	10	0.12	0.128

To demonstrate that cross-resistance was not generated, the MIC values of Nu-3 were evaluated against bacteria that had developed resistance to ciprofloxacin at day 21 of the serial passage experiment. The resulting MIC values of ≤5 mg/mL exhibited only slight differences from the initial measurements of 10 mg/mL (Table S4).

### *In vivo* activity

**Fig 5:**
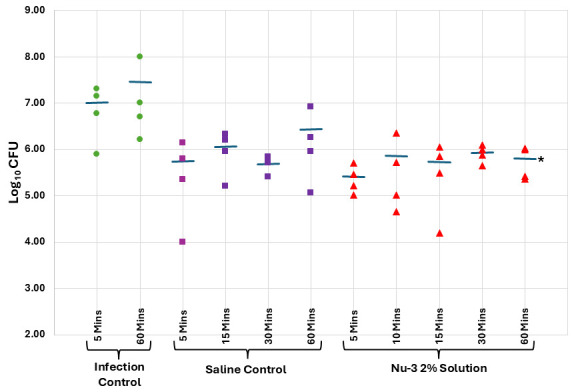
Results from the murine acute dermal infection with MRSA using a 2% solution of Nu-3 as the active, compared to infection and vehicle (saline) controls as described in the Materials and Methods. *, significantly different from infection control as calculated using ANOVA at 95% confidence; bars, mean of observations.

**Fig 6:**
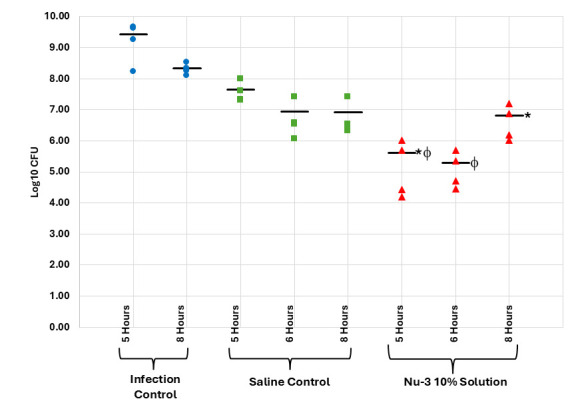
Results from the single dose murine acute dermal infection with MRSA using a 10% solution of Nu-3 as the active, compared to infection and vehicle (saline) controls as described in the Materials and Methods. *, significantly different from infection control as calculated using ANOVA at 95% confidence; ϕ, significantly different from saline control as calculated using ANOVA at 95% confidence; bars, mean of observations.

Murine models of acute dermal infection are used as a surrogate for diabetic foot ulcer infections, and Nu-3 was profiled in these models to provide evidence that the compound can combat skin infections *in vivo*. Skin infections were induced by application of a bacterial inoculum of 10^6^ CFU to abraded skin and then allowing the infection to become established for 4 h before treatments were initiated. Solution formulations of Nu-3 at two different concentrations—2% and 10%—and a pH value of 1.5 were tested against *S. aureus* (USA300)—a community-acquired MRSA (14), where each treatment group comprised four animals. The 2% solution demonstrated efficacy at 60 min post-treatment with a 1.2 log reduction in CFU (97.8% reduction) (see Fig. 5; Table S5), and the 10% solution demonstrated robust activity at 1, 2, and 4 h post-treatment with a 3.83 log reduction in CFU at 1 h post-treatment (>99.99% reduction) and a 1.52 log reduction at 4 h post-treatment in CFU (96.9% reduction) (see Fig. 6; Table S6). In contrast, a 10% solution formulation at a pH value of 4 was ineffective in treating the USA300 dermal infection (data not shown), and all subsequent experiments were conducted with formulations at pH 1.5.

**Fig 7:**
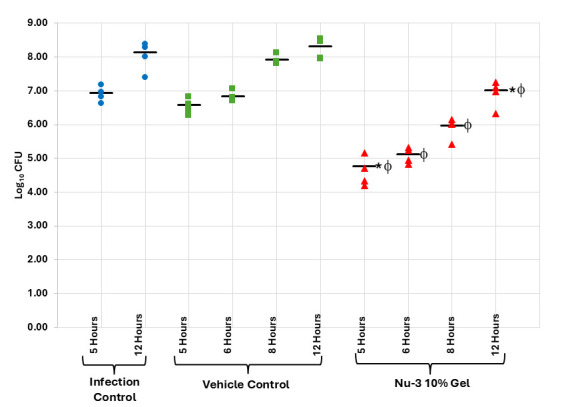
Results from the single dose murine acute dermal infection with MRSA using a 10% gel of Nu-3 as the active, compared to infection and vehicle (gel) controls as described in the Materials and Methods. *, significantly different from infection control as calculated using ANOVA at 95% confidence; ϕ, significantly different from vehicle control as calculated using ANOVA at 95% confidence; bar, mean of observations.

**Fig 8:**
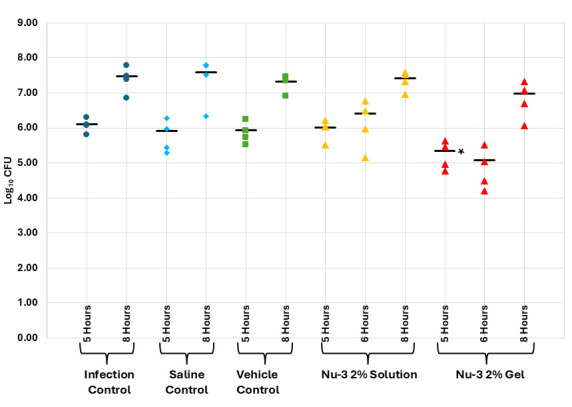
Results from the single dose murine acute dermal infection with MRSA comparing a 2% solution and 2% gel of Nu-3, as the active treatments, to infection, saline, and vehicle gel controls as described in the Materials and Methods. *, significantly different from infection control as calculated using ANOVA at 95% confidence; bar, mean of observations.

In a second step, the Nu-3 gel formulation was profiled. Topical application of a 10% gel to an established infection displayed robust activity compared to both untreated controls and placebo gel formulation at 1, 2, 4, and 8 h post-treatment (where each treatment group comprised four animals), with a 2.17 log reduction in CFU at 1 h post-treatment (99.3% reduction) and a 1.11 log reduction in CFU at 8 h post-treatment (92.3% reduction) (see Fig. 7; Table S7). In a comparative study of 2% solution versus 2% gel (where each treatment group comprised four animals), the gel formulation was better than the solution formulation at all time points but was only significantly better than the vehicle and saline controls at 1 h post-treatment with a 0.76 log reduction in CFU (82.7% reduction) (see Fig. 8; Table S8).

**Fig 9:**
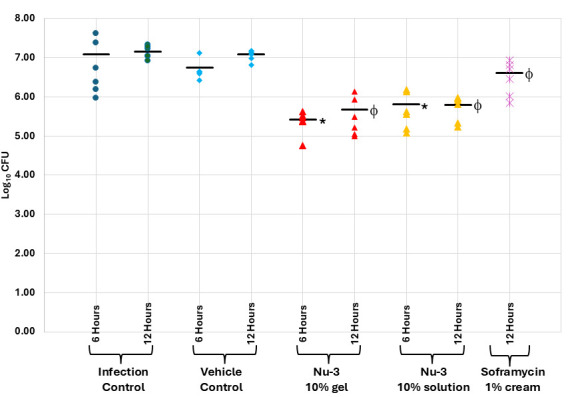
Results from the single dose murine acute dermal infection with MRSA comparing 10% solution of Nu-3, 10% gel of Nu-3, and 1% soframycin cream as the active treatments, to infection and vehicle gel controls as described in the Materials and Methods. *, significantly different from infection control at 6 h as calculated using ANOVA at 95% confidence; ϕ, significantly different from infection control at 12 h as calculated using ANOVA at 95% confidence; bar, mean of observations.

In a further set of experiments, skin infections were induced by application of a 10^6^ CFU inoculum of *S. aureus* (ATCC 43300) to tape-stripped skin and then the infection was allowed to become established for 4 h. Using this protocol, a 10% solution formulation of Nu-3 was compared to a 10% gel formulation and 1% Soframycin cream as an active control, at 2 and 8 h post-treatment, where each treatment group comprised six animals. The gel formulation was better than the solution formulation, with the gel showing a 1.67 log reduction (97.9% reduction) of CFU compared to vehicle at 2 h post-treatment and a 1.48 log reduction (96.7% reduction) of CFU compared to vehicle, and both treatments were superior to the active control Soframycin**,** which showed a 0.6 log reduction in CFU compared to the infection control (see Fig. 9; Table S9).

**Fig 10:**
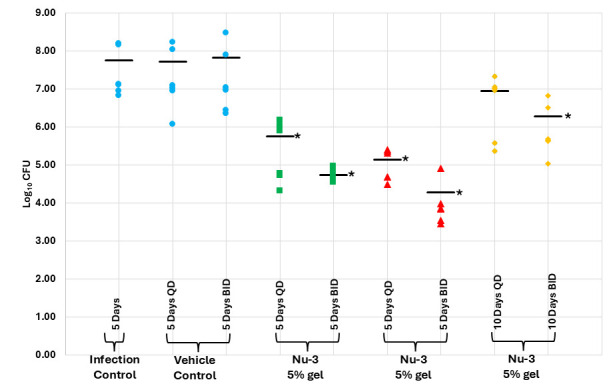
Results from the multiple dose murine acute dermal infection with MRSA comparing 5% and 10% Nu-3 gels to infection and vehicle gel controls at 5 days and 10 days post-infection as described in the Materials and Methods. *, significantly different from infection control at 5 days as calculated using ANOVA at 95% confidence; bar, mean of observations.

To address the duration of antibiotic effect, 5-day multiple-dose applications of the 5% and 10% gel formulations were studied using both QD and BID regimens, where each treatment group comprised six animals. As before, the 10% formulation was superior to the 5% formulation when administered either QD or BID, demonstrating a 3.43 log reduction in CFU in the BID regimen (>99.9% reduction) and a 2.69 log reduction in CFU in the QD regimen (99.8% reduction) after 5 days of dosing. In addition, the BID regimen outperformed the QD administration, where the 5% gel showed log reductions in CFUs of 1.79 and 2.07 compared to vehicle control (98.4% and 99.1% reductions, respectively) after 5 days of dosing. As an extension to the study, the animals in the 5% gel QD and BID treatment groups were allowed to recover for an additional 5 days without treatment, and as expected, the BID regimen was improved over the QD regimen, where the BID regimen showed a 1.43 log reduction in CFU (96.3% reduction) and the QD regimen showed a 0.88 log reduction in CFU (86.7% reduction, see Fig. 10; Table S10).

## DISCUSSION

DFUs represent a complex and difficult-to-treat complication of diabetes mellitus and include three major pathologic conditions that appear as the main contributors to the development of DFUs: ischemia, neuropathy, and infection. Infection control is accepted to be a key success factor in facilitating healing and preventing disease progression to a more severe form of infection, and up to a fifth of diabetic patients who have a foot ulcer will need amputation (15). Accordingly, infections, if not contained, are an important additional risk factor alongside the presence of peripheral arterial disease for future amputations (16–18), and this underlines the important role of an early and effective treatment of the infection in an early stage to prevent disease progression.

The current standard of care for the treatment of an infection in DFU is described in guidelines issued in 2016 (19), as well as more recently in 2023 (20), and includes the use of oral antibiotics, although there is a paucity of controlled clinical data that demonstrates their effectiveness. A clear impediment to the activity of systemic antibiotics against foot ulcer infections is the poor perfusion to the wound area as a result of microvascular disease, and thereby the difficulty to attain drug concentrations greater than the MICs. Conditions like mild infections of more superficial diabetic foot ulcers with poor perfusion may accordingly benefit from the application of an antimicrobial drug through local drug delivery. This would permit achieving a high and sufficient antimicrobial activity directly applied to the infected wound while avoiding most of the side effects related to systemic exposure by oral or intravenous antibiotics, which have a questionable and insufficiently documented effect and benefit in the treatment of infected DFUs.

Nu-3 is a member of a new class of antimicrobials, the Bisphosphocin class, which combines all the potential benefits of an antimicrobial well-suited for skin infections and specifically mildly infected diabetic foot ulcers. Thus, Nu-3 displays a broad antibacterial spectrum, efficacy against resistant pathogens, low susceptibility to resistant development, and topical drug delivery as a preferred therapeutic approach in these conditions. In a previous study, the Bisphosphocin Nu-3 was shown to have antimicrobial activity against *S. aureus*, both susceptible and resistant strains, *P. aeruginosa*, *Burkholderia pseudomallei, Bacillus anthracis, Francisella tularensis,* and *Yersinia pestis* (6). In addition, previous studies have demonstrated that Nu-3 exhibited strong bactericidal activity against *A. baumannii* and *Staphylococcus epidermidis* that were encapsulated within a biofilm layer on the surface of a borosilicate glass tube (21). Nu-3 has now been profiled against an extended battery of bacterial species, and the studies show that Nu-3 displays a very broad spectrum of antibiotic activity covering Gram-positive, Gram-negative, and a diverse range of resistant organisms that include all the major bacteria isolated from infections in diabetic foot ulcers with MICs in the range of 0.78 to 25 mg/mL. Importantly, Nu-3 was also tested against a range of recent clinical isolates (2022) from diabetic foot infections, and from other wounds, which included susceptible and resistant strains of *S. aureus* and *E. faecalis*, confirming its antibacterial activity and its potential as an antibiotic for the treatment of infections in DFU. A recent meta-analysis reporting that resistant pathogens can be identified in 15%–20% of iDFUs underscores the urgent need for a highly potent topical treatment targeting these pathogens (22). Currently, no such treatment is available.

Nu-3 shows a rapid, cidal action at pH 1.5 and 3.5 at similar concentrations with complete eradication of the diabetic foot ulcer infection-relevant bacteria *K. pneumoniae, P. mirabilis, A. baumannii, E. coli, P. aeruginosa, S. aureus,* and *S. saprophyticus*, which occurs within minutes and at concentrations that are being targeted for the clinical trial of Nu-3 (5% and 10% equivalent to 50 and 100 mg/mL, respectively).

Attempts to generate resistance against Nu-3 in *S. aureus* and *E. coli* using a 21-day serial passage assay were unsuccessful and indicate that the use of Nu-3 is unlikely to induce resistance. Importantly, the positive control in the resistance experiments, ciprofloxacin, displayed significant resistance at day 21 for both bacteria, and importantly, the ciprofloxacin-resistant bacteria were still susceptible to Nu-3 at the typical MIC of Nu-3, indicating the mechanism of the ciprofloxacin resistance did not impede the activity of Nu-3. Nu-3 displayed similar activities across all bacteria, both Gram-positive and Gram-negative, irrespective of their antibiotic resistance markers, indicating a greatly reduced susceptibility to the established forms of resistance. Importantly, although the MIC values of Nu-3 are higher than those of most antibiotics, the Bisphosphocin class is being actively pursued because of the excellent *in vivo* tolerance exhibited by the series, the low probability of resistance development, rapid bacterial eradication within minutes (1–10 min), and the ability to administer doses that cover the MICs in a topical application.

The *in vitro* profile of Nu-3 is an excellent match for the treatment of infections in diabetic foot ulcers, and the ability of Nu-3 to treat dermal infections in mice was confirmed. Additional studies to profile Nu-3 in diabetic animals, with and without ischemic or neuropathic conditions, as well as in polymicrobial infections, will be conducted in the future. The topical route was selected since it enables the use of higher concentrations at the site of infection and minimizes any systemic side effects, subject to any toxicological findings. A preliminary round of testing was conducted with a solution formulation, where the degree of efficacy was found to be dose-dependent, with greater reductions in CFU at higher concentrations of Nu-3 solution. Nu-3 is an acidic molecule, and initial testing was conducted at pH 1.5, which is close to the pKa of the molecule. However, testing at pH 4 failed to show any significant activity, which is attributed to a lower concentration of the free acid form of Nu-3. This may indicate that any wounds that have high volumes of exudate, which are typically at a higher pH, could lead to buffering of the Nu-3 formulation and a reduction in potency and indicates that large volumes of exudate at pH 4–6 could reduce the activity or neutralize the activity of Nu-3.

The solution formulation tended to have a shorter duration of action, where the infection re-emerged within 4 h of the treatment in single-dose experiments. Additionally, application of a solution form of Nu-3, especially to diabetic foot ulcers on the plantar side of the foot, would more likely be dispersed quickly. For that reason, a gel formulation was developed with the expectation that the gel form would have improved retention characteristics at the site of infection/application. In the murine dermal skin infection models, the gel formulation showed improved activity over the solution formulation in terms of potency (log_10_ CFU reduction and % reduction) at every time point and also displayed an improved duration of action, where re-emergence of the infection was noticed only after 8 h post-single dose treatment. As with the solution formulation, the gel formulation showed a dose-dependent increase in activity from 2% to 5% and to 10%.

Given the re-emergence of infections in the single-dose models and the probable clinical protocol for treatment of infections in diabetic foot ulcers, Nu-3 was profiled in a multidose experiment, and both 5% and 10% gels were clearly efficacious, where a BID regimen was improved over the QD regimen. Extension of the model observation to include a 5-day “recovery” period after the completion of the 5-day treatment period, where no active drug was applied to the wound, also indicated that a multidose regimen is more effective at clearing infections than a single dose.

In conclusion, the *in vitro* and *in vivo* studies reported here suggest that Nu-3 gel has the ideal characteristics to be used in the treatment of mild infections in diabetic foot ulcers—a broad antibacterial spectrum, including Gram-positive, Gram-negative, and multidrug-resistant bacteria, rapid cidal action, the ability to be applied topically at relatively high concentrations, a low probability for resistance development, and clear efficacy in murine models of skin infection. Accordingly, Nu-3 is being advanced into clinical development.

## SUPPLEMENTAL MATERIAL

Supplemental material aac.00948-25-s0001.docx MIC protocol; Tables S1 to S10.

ASM does not own the copyrights to Supplemental Material that may be linked to, or accessed through, an article. The authors have granted ASM a non-exclusive, world-wide license to publish the Supplemental Material files. Please contact the corresponding author directly for reuse.

## References

[1] Murray CJL, Ikuta KS, Sharara F, Swetschinski L, Robles Aguilar G, Gray A, Han C, Bisignano C, Rao P, Wool E, et al.. 2022. Global burden of bacterial antimicrobial resistance in 2019: a systematic analysis. The Lancet 399:629–655. doi:10.1016/S0140-6736(21)02724-0 PMC8841637 35065702

[2] U.S. Department of Health and Human Services Center for Disease Control. 2019. Antibiotic resistance threats in the United States

[3] World Bank. 2017. “Drug-resistant infections: a threat to our economic future.” License: Creative Commons Attribution CC BY 3.0 IGO. World Bank, Washington, DC.

[4] Nelson RE, Hatfield KM, Wolford H, Samore MH, Scott RD, Reddy SC, Olubajo B, Paul P, Jernigan JA, Baggs J. 2021. National estimates of healthcare costs associated with multidrug-resistant bacterial infections among hospitalized patients in the United States. Clin Infect Dis 72:S17–S26. doi:10.1093/cid/ciaa1581 33512523 PMC11864165

[5] Lin Q, Pilewski JM, Di YP. 2021. Acidic microenvironment determines antibiotic susceptibility and biofilm formation of Pseudomonas aeruginosa. Front Microbiol 12:747834. doi:10.3389/fmicb.2021.747834 34867864 PMC8640179

[6] Lipsky BA, Berendt AR, Cornia PB, Pile JC, Peters EJG, Armstrong DG, Deery HG, Embil KM, Jospeh WS, Karchmer AW, Pinzur MS, Senneville E. 2012. Executive Summary: 2012 Infectious Diseases Society of America clinical practice guideline for the diagnosis and treatment of diabetic foot infections. Clin Infect Dis 54:132–173. doi:10.1093/cid/cis34 22619239

[7] Sadeghpour Heravi F, Zakrzewski M, Vickery K, G Armstrong D, Hu H. 2019. Bacterial diversity of diabetic foot ulcers; current status and future prospectives. J Clin Med 8:1935. doi:10.3390/jcm8111935 31717640 PMC6912738

[8] Chai W, Wang Y, Zheng H, Yue S, Liu Y, Wu Y, Li X. 2021. The profile of microbiological pathogens in diabetic foot ulcers. Front Med 8. doi:10.3389/fmed.2021.656467 PMC8491778 34621756

[9] Cao S, Sun LQ, Wang M. 2011. Antimicrobial activity and mechanism of action of Nu-3, a protonated modified nucleotide. Ann Clin Microbiol Antimicrob 10:1–9. doi:10.1186/1476-0711-10-1 21232163 PMC3031213

[10] Wong JP, DiTullio P, Parkinson S. 2015. Bisphosphocins: novel antimicrobials for enhanced killing of drug-resistant and biofilm-forming bacteria. Future Microbiol 10:1751–1758. doi:10.2217/fmb.15.70 26597426

[11] 2018. Nu-3 is supplied as a disodium salt at >95% purity as characterized by 1H-,13C- and 35P-nmr, mass spectroscopy, and HPLC mass spec

[12] Clinical and Laboratory Standards Institute (CLSI). 2018. Methods for dilution antimicrobial susceptibility tests for bacteria that grow aerobically; Approved Standard—Eleventh Edition. Clinical and Laboratory Standards Institute document M07. Clinical and Laboratory Standards Institute, 950 West Valley Road, Suite 2500, Wayne, Pennsylvania 19087, USA. In CLSI. Performance Standards for Antimicrobial Susceptibility Testing, 32nd Ed. CLSI document M100, Wayne, Pennsylvania 19087, USA.

[13] Committee for the purpose of control and supervision on experiments on animals. 2003. CPCSEA guidelines for laboratory animal facility. Indian J Pharmacol 35:257–274.

[14] Carrel M, Perencevich EN, David MZ. 2015. USA300 methicillin-resistant Staphylococcus aureus, United States, 2000-2013. Emerg Infect Dis 21:1973–1980. doi:10.3201/eid2111.150452 26484389 PMC4622244

[15] Lipsky BA, Berendt AR, Cornia PB, Pile JC, Peters EJG, Armstrong DG, Deery HG, Embil JM, Joseph WS, Karchmer AW, Pinzur MS, Senneville E, Infectious Diseases Society of America. 2012. 2012 Infectious Diseases Society of America clinical practice guideline for the diagnosis and treatment of diabetic foot infections. Clin Infect Dis 54:e132–e173. doi:10.1093/cid/cis346 22619242

[16] Armstrong DG, Lavery LA, Harkless LB. 1998. Validation of a diabetic wound classification system. The contribution of depth, infection, and ischemia to risk of amputation. Diabetes Care 21:855–859. doi:10.2337/diacare.21.5.855 9589255

[17] Oyibo SO, Jude EB, Tarawneh I, Nguyen HC, Harkless LB, Boulton AJ. 2001. A comparison of two diabetic foot ulcer classification systems: the Wagner and the University of Texas wound classification systems. Diabetes Care 24:84–88. doi:10.2337/diacare.24.1.84 11194247

[18] Jia L, Parker CN, Parker TJ, Kinnear EM, Derhy PH, Alvarado AM, Huygens F, Lazzarini PA, Diabetic Foot Working Group, Queensland Statewide Diabetes Clinical Network (Australia). 2017. Incidence and risk factors for developing infection in patients presenting with uninfected diabetic foot ulcers. PLoS One 12:e0177916. doi:10.1371/journal.pone.0177916 28545120 PMC5435321

[19] Lipsky BA, Aragón-Sánchez J, Diggle M, Embil J, Kono S, Lavery L, Senneville E, Urbancic-Rovan V, Asten S, Peters EJG. 2016. International Working Group on the Diabetic Foot (IWGDF) guidance on the diagnosis and management of foot infections in persons with diabetes10.1002/dmrr.2699 26386266

[20] Senneville É, Albalawi Z, van Asten SA, Abbas ZG, Allison G, Aragón-Sánchez J, Embil JM, Lavery LA, Alhasan M, Oz O, Uçkay I, Urbančič-Rovan V, Xu Z-R, Peters EJG. 2023. IWGDF/IDSA guidelines on the diagnosis and treatment of diabetes-related foot infections (IWGDF/IDSA 2023). Clin Infect Dis. doi:10.1093/cid/ciad527 37779457

[21] Akiyoshi D, Dilo J, DiTullio P. 2013. Novel Bisphosphocin Nu-3 demonstrates rapid killing of bacteria-encased in biofilm in vitro, 2013, 53rd. ICAAC.

[22] Zhou S, Hu X, Wang Y, Fei W, Sheng Y, Que H. 2024. The global prevalence of methicillin-resistant Staphylococcus aureus in patients with diabetic foot ulcers: a systematic review and meta-analysis. Diabetes Metab Syndr Obes 17:563–574. doi:10.2147/DMSO.S446911 38333763 PMC10849909

