# Supplementary material for: In vitro and in vivo characterization of Bisphosphocin Nu-3—a novel broad-spectrum antimicrobial compound with high potency against resistant pathogens
Source: Antimicrob Agents Chemother. 2025 Oct 21;69(12):e00948-25. doi: 10.1128/aac.00948-25 (PMC12691638; doi:10.1128/aac.00948-25)
Supplement: Supplemental material — MIC protocol; Tables S1 to S10. [file aac.00948-25-s0001.docx]

**Supplementary Information.**

**MIC Test protocol**

Test Media

Cation-adjusted Mueller Hinton broth (CAMHB; BD; Lot No.1242967) was used for MIC testing of aerobic organisms. For Streptococcus and Corynebacterium isolates, this medium was supplemented with 3% laked horse blood (LHB; Hemostat; Dixon, CA; Lot No. 652081). For H. influenzae, Haemophilus Test medium (HTM) was used. HTM was made by supplementing CAMHB with 15 µg/mL nicotinamide adenine dinucleotide (NAD; Sigma; St. Louis, MO; Lot. No. W58778), 15 µg/mL hematin porcine (Sigma; Lot No. SLBD4979V) and 5 g/L of yeast extract (Sigma; Lot No. SLBX6973).

Broth Microdilution MIC Methodology

MIC values were determined using a broth microdilution procedure described by CLSI (1, 3). Ninety-six-well MIC plates were prepared using automated liquid handlers for serial dilutions and liquid transfers. Automated liquid handlers included the Multidrop 384 (Labsystems, Helsinki, Finland) and the Biomek 3000 (Beckman Coulter, Fullerton CA).

All wells in columns 2 through 12 of a deep-well polypropylene plate (Costar 3879; polypropylene) were filled with 1 mL of the appropriate diluent. Then, 2 mL of the tested agents were added to the wells of column 1 of the plates at 10X the highest final concentration to be tested. Serial two-fold dilutions were made across the rows through column 11 by hand. The wells of column 12 contained no drug and served as the growth control wells.

The daughter plates (Greiner 650162; polystyrene) were then created using the Biomek FX which transferred 100 µL of 2X drug solution from each well of the 2X deep well polypropylene mother plate to each corresponding well of the daughter plate in a single step. A standardized inoculum of each test organism was prepared per CLSI methods (1) to equal a 0.5 McFarland standard into 0.9% sterile saline, followed by an additional 1:10 dilution in saline. The plates were then inoculated with 10 μL of the inoculum suspension using the Biomek 3000 from low to high drug concentration, resulting in a final concentration of approximately 5 x 105 CFU/mL per well for bacteria.

After this transfer the plates were stacked 3 to 4 high, covered with a sterile lid on the top plate, and incubated aerobically at 35ºC. After 30-minutes incubation the plates were removed from the incubator and the Multidrop 384 was used to add an additional 90 μL of 2.1X of appropriate test media to reach the final assay volume of ~0.2 mL.

Alongside this nonstandard testing methodology, a standard MIC was conducted for QC organisms and comparator drugs. The wells in columns 2 through 12 of a standard 96-well microdilution plate were filled with 150 μL of the appropriate diluent. Three hundred microliters of each comparator stock solution at 100X the highest concentration to be tested was added to one well in column 1 of the plates. The Biomek 3000 was used to complete serial 2-fold dilutions through column 11. The wells of column 12 contained no drug and served as the organism Micromyx Study Report 09-15-2022-Lakewood 13 Page 4 of 11 growth control wells. These panels were the comparator mother plates. The daughter plates were loaded with 190 μL of the designated test medium using the Multidrop 384. The daughter plates were prepared on the Biomek FX instrument which transferred 2 μL of drug solution from each well of a mother plate to the corresponding well of each daughter plate in a single step. The wells of the daughter plates ultimately contained 190 μL of broth appropriate to the organism, 2 μL of drug solution, and 10 μL of test organism inoculum as described below.

A standardized inoculum of each test organism was prepared per CLSI methods (1, 3) to equal a 0.5 McFarland standard in CAMHB, followed by an additional 1:10 (1:100 for yeast) dilution. The plates were then inoculated with 10 μL of the inoculum suspension using the Biomek 3000 from low to high drug concentration, resulting in a final concentration of approximately 5 x 105 CFU/mL for bacteria and approximately 0.5 to 2.5 x 103 CFU/mL for yeast per well. Plates were stacked 3 to 4 high, covered with a lid on the top plate, placed in plastic bags, and incubated at 35ºC for approximately 16 to 20 hr (aerobes), 20 to 24 hr (Streptococcus and Haemophilus), 24-48 hr (Corynebacterium) 24 and 48 hr (yeast). Following incubation, the microplates were removed from the incubator and viewed from the bottom using a plate viewer. For each of the test media and each drug, an un-inoculated solubility control plate was observed for evidence of drug precipitation. The MIC was read and recorded as the lowest concentration of drug that inhibited visible growth of the organism.

Determination of pH in MIC Wells

Before and after incubation 10 μL aliquots were taken from the wells of the mother plate for preincubation pH and uninoculated solubility daughter plates for post-incubation pH and spotted onto an MColorpHast pH indicator strip. The pH of the sampled wells was determined by eye. Samples were taken from plates to determine the change in pH due to the acidity of the compounds. Comparators included antibiotics used in testing as well as HCl (pH 1.5) and citric acid (pH 3.5), which had been used to adjust the pH of Nu3 and Nu8, to those pH values, respectively.

**Table S1**: Detailed information on the recent clinical isolates tested and presented in Table 1 of the manuscript.

| Collection number | Species | Category | US census division | State | Age | Gender | Specimen type |
| --- | --- | --- | --- | --- | --- | --- | --- |
| 1231079 | *Enterococcus faecalis* | VSE | 8: Mountain | UT | 46 | F | Wound/Drainage/Ulcer |
| 1231150 | *Enterococcus faecalis* | VSE | 6: East South Central | KY | 83 | M | Wound/Drainage/Ulcer |
| 1244757 | *Enterococcus faecalis* | VSE | 5: South Atlantic | VA | 69 | M | Diabetic foot infection |
| 1244798 | *Enterococcus faecalis* | VSE | 5: South Atlantic | VA | 79 | M | Diabetic foot infection |
| 1235228 | *Enterococcus faecalis* | VRE | 7: West South Central | TX | 56 | M | Wound/Drainage/Ulcer |
| 1265416 | *Enterococcus faecalis* | VRE | 2: Middle Atlantic | NY | 83 | M | Wound/Drainage/Ulcer |
| 1230647 | *Staphylococcus aureus* | MSSA | 8: Mountain | UT | 31 | M | Diabetic foot infection |
| 1231136 | *Staphylococcus aureus* | MSSA | 6: East South Central | KY | 36 | F | Diabetic foot infection |
| 1234299 | *Staphylococcus aureus* | MSSA | 3: East North Central | MI | 63 | F | Diabetic foot infection |
| 1236804 | *Staphylococcus aureus* | MSSA | 4: West North Central | NE | 79 | F | Diabetic foot infection |
| 1244470 | *Staphylococcus aureus* | MSSA | 7: West South Central | TX | 74 | M | Diabetic foot infection |
| 1249032 | *Staphylococcus aureus* | MSSA | 5: South Atlantic | VA | 73 | F | Diabetic foot infection |
| 1231131 | *Staphylococcus aureus* | MRSA | 6: East South Central | KY | 57 | M | Diabetic foot infection |
| 1232605 | *Staphylococcus aureus* | MRSA | 9: Pacific | WA | 59 | M | Wound/Drainage/Ulcer |
| 1233808 | *Staphylococcus aureus* | MRSA | 6: East South Central | KY | 45 | M | Wound/Drainage/Ulcer |
| 1244473 | *Staphylococcus aureus* | MRSA | 7: West South Central | TX | 64 | M | Diabetic foot infection |
| 1244794 | *Staphylococcus aureus* | MRSA | 5: South Atlantic | VA | 64 | M | Diabetic foot infection |
| 1249449 | *Staphylococcus aureus* | MRSA | 4: West North Central | IA |  |  |  |
| 1250232 | *Staphylococcus aureus* | MRSA | 3: East North Central | IN | 1 | M | Abscess (pus) |
| 1256700 | *Staphylococcus aureus* | MRSA | 3: East North Central | WI | 1 | M | Abscess (pus) |
| 1265381 | *Staphylococcus aureus* | MRSA | 2: Middle Atlantic | NY | 61 | M | Wound/Drainage/Ulcer |
| 1270046 | *Staphylococcus aureus* | MRSA | 5: South Atlantic | FL | 91 | F | Diabetic foot infection |

Abbreviations: F, female; M, male; MRSA, methicillin-resistant *Staphylococcus aureus*; MSSA, methicillin-susceptible *Staphylococcus aureus*; VRE, vancomycin-resistant *enterococci*; VSE, vancomycin-susceptible *enterococci.*

**Table S2:** Detailed data for Nu-3 concentrations in mg/mL required to demonstrate complete kill of six strains of bacteria at 1 and 10 minutes.

| Compound | Species | Code | pH | Concentration (mg/ml) | Time (min) | Density % |
| --- | --- | --- | --- | --- | --- | --- |
| Nu-03 | A. baumannii | ATCC-19606 | 1.5 | 1.25 | 1 | 100 |
| Nu-03 | A. baumannii | ATCC-19606 | 1.5 | 2.5 | 1 | 100 |
| Nu-03 | A. baumannii | ATCC-19606 | 1.5 | 5 | 1 | 100 |
| Nu-03 | A. baumannii | ATCC-19606 | 1.5 | 10 | 1 | 50 |
| Nu-03 | A. baumannii | ATCC-19606 | 1.5 | 20 | 1 | 0 |
| Nu-03 | A. baumannii | ATCC-19606 | 1.5 | 1.25 | 10 | 100 |
| Nu-03 | A. baumannii | ATCC-19606 | 1.5 | 2.5 | 10 | 0 |
| Nu-03 | A. baumannii | ATCC-19606 | 1.5 | 5 | 10 | 0 |
| Nu-03 | A. baumannii | ATCC-19606 | 1.5 | 10 | 10 | 0 |
| Nu-03 | A. baumannii | ATCC-19606 | 1.5 | 20 | 10 | 0 |
| Nu-03 | E. coli | UTI89 | 1.5 | 1.25 | 1 | 100 |
| Nu-03 | E. coli | UTI89 | 1.5 | 2.5 | 1 | 100 |
| Nu-03 | E. coli | UTI89 | 1.5 | 5 | 1 | 100 |
| Nu-03 | E. coli | UTI89 | 1.5 | 10 | 1 | 100 |
| Nu-03 | E. coli | UTI89 | 1.5 | 20 | 1 | 100 |
| Nu-03 | E. coli | UTI89 | 1.5 | 1.25 | 10 | 100 |
| Nu-03 | E. coli | UTI89 | 1.5 | 2.5 | 10 | 100 |
| Nu-03 | E. coli | UTI89 | 1.5 | 5 | 10 | 100 |
| Nu-03 | E. coli | UTI89 | 1.5 | 10 | 10 | 0 |
| Nu-03 | E. coli | UTI89 | 1.5 | 20 | 10 | 0 |
| Nu-03 | P. aeruginosa | ATCC-27853 | 1.5 | 1.25 | 1 | 100 |
| Nu-03 | P. aeruginosa | ATCC-27853 | 1.5 | 2.5 | 1 | 80 |
| Nu-03 | P. aeruginosa | ATCC-27853 | 1.5 | 5 | 1 | 0 |
| Nu-03 | P. aeruginosa | ATCC-27853 | 1.5 | 10 | 1 | 0 |
| Nu-03 | P. aeruginosa | ATCC-27853 | 1.5 | 20 | 1 | 0 |
| Nu-03 | P. aeruginosa | ATCC-27853 | 1.5 | 1.25 | 10 | 0 |
| Nu-03 | P. aeruginosa | ATCC-27853 | 1.5 | 2.5 | 10 | 0 |
| Nu-03 | P. aeruginosa | ATCC-27853 | 1.5 | 5 | 10 | 0 |
| Nu-03 | P. aeruginosa | ATCC-27853 | 1.5 | 10 | 10 | 0 |
| Nu-03 | P. aeruginosa | ATCC-27853 | 1.5 | 20 | 10 | 0 |
| Nu-03 | S. saprophyticus | ATCC-15305 | 1.5 | 1.25 | 1 | 100 |
| Nu-03 | S. saprophyticus | ATCC-15305 | 1.5 | 2.5 | 1 | 50 |
| Nu-03 | S. saprophyticus | ATCC-15305 | 1.5 | 5 | 1 | 0 |
| Nu-03 | S. saprophyticus | ATCC-15305 | 1.5 | 10 | 1 | 0 |
| Nu-03 | S. saprophyticus | ATCC-15305 | 1.5 | 20 | 1 | 0 |
| Nu-03 | S. saprophyticus | ATCC-15305 | 1.5 | 1.25 | 10 | 0 |
| Nu-03 | S. saprophyticus | ATCC-15305 | 1.5 | 2.5 | 10 | 0 |
| Nu-03 | S. saprophyticus | ATCC-15305 | 1.5 | 5 | 10 | 0 |
| Nu-03 | S. saprophyticus | ATCC-15305 | 1.5 | 10 | 10 | 0 |
| Nu-03 | S. saprophyticus | ATCC-15305 | 1.5 | 20 | 10 | 0 |
| Nu-03 | K. pneumoniae | ATCC-33495 | 1.5 | 1.25 | 1 | 100 |
| Nu-03 | K. pneumoniae | ATCC-33495 | 1.5 | 2.5 | 1 | 100 |
| Nu-03 | K. pneumoniae | ATCC-33495 | 1.5 | 5 | 1 | 50 |
| Nu-03 | K. pneumoniae | ATCC-33495 | 1.5 | 10 | 1 | 0 |
| Nu-03 | K. pneumoniae | ATCC-33495 | 1.5 | 20 | 1 | 0 |
| Nu-03 | K. pneumoniae | ATCC-33495 | 1.5 | 1.25 | 10 | 80 |
| Nu-03 | K. pneumoniae | ATCC-33495 | 1.5 | 2.5 | 10 | 0 |
| Nu-03 | K. pneumoniae | ATCC-33495 | 1.5 | 5 | 10 | 0 |
| Nu-03 | K. pneumoniae | ATCC-33495 | 1.5 | 10 | 10 | 0 |
| Nu-03 | K. pneumoniae | ATCC-33495 | 1.5 | 20 | 10 | 0 |
| Nu-03 | P. mirabilis | ATCC-7002 | 1.5 | 1.25 | 1 | 100 |
| Nu-03 | P. mirabilis | ATCC-7002 | 1.5 | 2.5 | 1 | 100 |
| Nu-03 | P. mirabilis | ATCC-7002 | 1.5 | 5 | 1 | 100 |
| Nu-03 | P. mirabilis | ATCC-7002 | 1.5 | 10 | 1 | 100 |
| Nu-03 | P. mirabilis | ATCC-7002 | 1.5 | 20 | 1 | 100 |
| Nu-03 | P. mirabilis | ATCC-7002 | 1.5 | 1.25 | 10 | 100 |
| Nu-03 | P. mirabilis | ATCC-7002 | 1.5 | 2.5 | 10 | 100 |
| Nu-03 | P. mirabilis | ATCC-7002 | 1.5 | 5 | 10 | 100 |
| Nu-03 | P. mirabilis | ATCC-7002 | 1.5 | 10 | 10 | 0 |
| Nu-03 | P. mirabilis | ATCC-7002 | 1.5 | 20 | 10 | 0 |

**Table S3:** Detailed data for the time kill reduction of *E. coli* (ATCC-25922) CFU vs time for a range of Nu-3 concentrations at pH 3.5. (CFU= colony forming units).

| **Compound** | **Species** | **Species Code** | **pH** | **Concentration (mg/ml)** | **Time (min)** | **log 10 CFU** |
| --- | --- | --- | --- | --- | --- | --- |
| Nu-03 | E. coli | ATCC-25922 | 3.5 | 25 mg/mL | 0 | 5.25 |
| Nu-03 | E. coli | ATCC-25922 | 3.5 | 25 | 1 | 3.38 |
| Nu-03 | E. coli | ATCC-25922 | 3.5 | 25 | 3 | 2.55 |
| Nu-03 | E. coli | ATCC-25922 | 3.5 | 25 | 5 | 1.99 |
| Nu-03 | E. coli | ATCC-25922 | 3.5 | 25 | 10 | 0.00 |
| Nu-03 | E. coli | ATCC-25922 | 3.5 | 25 | 20 | 0.00 |
| Nu-03 | E. coli | ATCC-25922 | 3.5 | 50 mg/mL | 0 | 5.25 |
| Nu-03 | E. coli | ATCC-25922 | 3.5 | 50 | 1 | 3.00 |
| Nu-03 | E. coli | ATCC-25922 | 3.5 | 50 | 3 | 2.10 |
| Nu-03 | E. coli | ATCC-25922 | 3.5 | 50 | 5 | 0.00 |
| Nu-03 | E. coli | ATCC-25922 | 3.5 | 50 | 10 | 0.00 |
| Nu-03 | E. coli | ATCC-25922 | 3.5 | 50 | 20 | 0.00 |
| Nu-03 | E. coli | ATCC-25922 | 3.5 | 100 mg/mL | 0 | 5.25 |
| Nu-03 | E. coli | ATCC-25922 | 3.5 | 100 | 1 | 2.85 |
| Nu-03 | E. coli | ATCC-25922 | 3.5 | 100 | 3 | 0.00 |
| Nu-03 | E. coli | ATCC-25922 | 3.5 | 100 | 5 | 0.00 |
| Nu-03 | E. coli | ATCC-25922 | 3.5 | 100 | 10 | 0.00 |
| Nu-03 | E. coli | ATCC-25922 | 3.5 | 100 | 20 | 0.00 |
| VC | E. coli | ATCC-25922 | 3.5 | 0 | 0 | 5.25 |
| VC | E. coli | ATCC-25922 | 3.5 | 0 | 1 | 4.92 |
| VC | E. coli | ATCC-25922 | 3.5 | 0 | 3 | 4.92 |
| VC | E. coli | ATCC-25922 | 3.5 | 0 | 5 | 4.93 |
| VC | E. coli | ATCC-25922 | 3.5 | 0 | 10 | 4.91 |
| VC | E. coli | ATCC-25922 | 3.5 | 0 | 20 | 4.90 |

**Table S4:** MIC data for Nu-3 and ciprofloxacin against the cultures from the 21-day serial exposure to ciprofloxacin.

|  | MIC (mg/mL) | |
| --- | --- | --- |
| Species | Nu-3 | Ciprofloxacin |
| *E. coli* (ATCC-25922) | <5.0 | 0.128 |
| *S. aureus* (ATCC-43300, MRSA) | <5.0 | 0.128 |

**Table S5:** Detailed data from the murine acute dermal infection with MRSA using a 2% solution of Nu-3 as the active, compared to infection and vehicle (saline) controls.

|  |  |  | **Log_10_ CFU** | | | | | |
| --- | --- | --- | --- | --- | --- | --- | --- | --- |
| **Group** | **Treatment** | **Time** | **1** | **2** | **3** | **4** | **Mean** | **SD** |
| 1 | Infection control | 5 mins | 5.90 | 7.30 | 7.15 | 6.77 | 7.01 | 0.63 |
| 2 | Saline Control | 5 mins | 6.14 | 5.35 | 5.79 | 4.00 | 5.75 | 0.94 |
| 3 | 2% Solution | 5 mins | 5.70 | 5.46 | 5.02 | 5.21 | 5.42 | 0.30 |
| 3 | 2% Solution | 10 mins | 5.02 | 4.65 | 6.35 | 5.72 | 5.86 | 0.75 |
| 2 | Saline Control | 15 mins | 6.32 | 5.95 | 6.19 | 5.20 | 6.07 | 0.50 |
| 3 | 2% Solution | 15 mins | 5.49 | 6.05 | 5.85 | 4.19 | 5.73 | 0.84 |
| 2 | Saline Control | 30 mins | 5.70 | 5.40 | 5.84 | 5.70 | 5.69 | 0.19 |
| 3 | 2% Solution | 30 mins | 5.88 | 6.00 | 5.65 | 6.10 | 5.94 | 0.19 |
| 1 | Infection control | 60 mins | 6.21 | 6.70 | 7.00 | 8.00 | 7.46 | 0.76 |
| 2 | Saline Control | 60 mins | 6.26 | 5.95 | 6.91 | 5.06 | 6.44 | 0.77 |
| 3 | 2% Solution | 60 mins | 6.00 | 5.42 | 6.02 | 5.36 | 5.80 | 0.36 |

**Table S6:** Detailed data from the single dose murine acute dermal infection with MRSA using a 10% solution of Nu-3 as the active, compared to infection and vehicle (saline) controls.

|  |  |  | **Log_10_ CFU** | | | | | |
| --- | --- | --- | --- | --- | --- | --- | --- | --- |
| **Group** | **Treatment** | **Time** | **1** | **2** | **3** | **4** | **Mean** | **SD** |
| 1 | Infection control | 5 hours | 9.67 | 9.63 | 8.24 | 9.27 | 9.44 | 0.67 |
| 2 | Saline Control | 5 hours | 7.30 | 7.32 | 7.60 | 7.99 | 7.65 | 0.32 |
| 3 | 10% Solution | 5 hours | 4.19 | 4.43 | 6.03 | 5.70 | 5.61 | 0.91 |
| 2 | Saline Control | 6 hours | 6.53 | 6.57 | 6.07 | 7.42 | 6.94 | 0.56 |
| 3 | 10% Solution | 6 hours | 5.35 | 5.69 | 4.72 | 4.45 | 5.30 | 0.57 |
| 1 | Infection control | 8 hours | 8.26 | 8.33 | 8.53 | 8.10 | 8.33 | 0.18 |
| 2 | Saline Control | 8 hours | 7.41 | 6.33 | 6.53 | 6.38 | 6.92 | 0.51 |
| 3 | 10% Solution | 8 hours | 6.03 | 6.87 | 6.19 | 7.21 | 6.82 | 0.56 |

**Table S7:** Detailed data from the single dose murine acute dermal infection with MRSA using a 10% gel of Nu-3 as the active, compared to infection and vehicle (gel) controls.

|  |  |  | **Log10 CFU** | | | | | |
| --- | --- | --- | --- | --- | --- | --- | --- | --- |
| **Group** | **Treatment** | **Time** | **1** | **2** | **3** | **4** | **Mean** | **SD** |
| 1 | Infection control | 5 hours | 6.96 | 6.62 | 6.82 | 7.18 | 6.94 | 0.24 |
| 2 | Vehicle Control | 5 hours | 6.81 | 6.60 | 6.45 | 6.26 | 6.58 | 0.23 |
| 3 | 10% Gel | 5 hours | 4.20 | 4.33 | 5.17 | 4.70 | 4.77 | 0.44 |
| 2 | Vehicle Control | 6 hours | 6.80 | 6.70 | 7.06 | 6.73 | 6.85 | 0.16 |
| 3 | 10% Gel | 6 hours | 4.82 | 5.32 | 5.24 | 4.94 | 5.13 | 0.24 |
| 2 | Vehicle Control | 8 hours | 7.87 | 8.12 | 7.80 | 7.82 | 7.92 | 0.15 |
| 3 | 10% Gel | 8 hours | 6.02 | 5.41 | 6.14 | 6.03 | 5.97 | 0.33 |
| 1 | Infection control | 12 hours | 8.28 | 8.00 | 7.40 | 8.37 | 8.14 | 0.44 |
| 2 | Vehicle Control | 12 hours | 7.95 | 8.54 | 7.97 | 8.46 | 8.31 | 0.31 |
| 3 | 10% Gel | 12 hours | 7.26 | 6.97 | 6.33 | 7.10 | 7.02 | 0.41 |

**Table S8:** Detailed data from the single dose murine acute dermal infection with MRSA comparing a 2% solution and 2% gel of Nu-3, as the active treatments, to infection, saline. and vehicle gel controls.

|  |  |  | **Log10 CFU** | | | | | |
| --- | --- | --- | --- | --- | --- | --- | --- | --- |
| **Group** | **Treatment** | **Time** | **1** | **2** | **3** | **4** | **Mean** | **SD** |
| 1 | Infection control | 5 hours | 6.30 | 6.09 | 5.81 | 6.06 | 6.10 | 0.20 |
| 2 | Saline Control | 5 hours | 5.44 | 5.28 | 6.28 | 5.98 | 5.92 | 0.47 |
| 3 | Vehicle Control | 5 hours | 5.92 | 6.23 | 5.73 | 5.51 | 5.93 | 0.31 |
| 4 | 2% Solution | 5 hours | 6.22 | 5.51 | 6.04 | 6.02 | 6.01 | 0.31 |
| 5 | 2% Gel | 5 hours | 5.46 | 4.96 | 4.78 | 5.63 | 5.34 | 0.40 |
| 4 | 2% Solution | 6 hours | 6.78 | 5.15 | 5.98 | 6.49 | 6.41 | 0.71 |
| 5 | 2% Gel | 6 hours | 5.04 | 5.51 | 4.48 | 4.20 | 5.08 | 0.58 |
| 1 | Infection control | 8 hours | 7.78 | 7.38 | 6.84 | 7.48 | 7.48 | 0.39 |
| 2 | Saline Control | 9 hours | 7.80 | 6.34 | 7.78 | 7.52 | 7.60 | 0.69 |
| 3 | Vehicle Control | 10 hours | 6.90 | 7.34 | 7.40 | 7.46 | 7.32 | 0.25 |
| 4 | 2% Solution | 11 hours | 7.59 | 7.32 | 7.54 | 6.97 | 7.41 | 0.28 |
| 5 | 2% Gel | 12 hours | 7.08 | 6.70 | 6.07 | 7.32 | 6.99 | 0.55 |

**Table S9:**  Detailed data from the single dose murine acute dermal infection with MRSA comparing 10% solution of Nu-3, 10% gel of Nu-3, and 1% soframycin cream as the active treatments, to infection and vehicle gel controls.

|  |  |  | **Log_10_ CFU** | | | | | | | |
| --- | --- | --- | --- | --- | --- | --- | --- | --- | --- | --- |
| **Group** | **Treatment** | **Time** | **1** | **2** | **3** | **4** | **5** | **6** | **Mean** | **SD** |
| 1 | Infection control | 6 hours | 7.37 | 7.61 | 6.73 | 5.96 | 6.19 | 6.37 | 7.09 | 0.66 |
| 2 | Vehicle Control | 6 hours | 6.63 | 6.64 | 6.43 | 6.64 | 6.60 | 7.13 | 6.74 | 0.24 |
| 3 | 10% Gel | 6 hours | 4.76 | 5.37 | 5.39 | 5.63 | 5.47 | 5.52 | 5.42 | 0.31 |
| 4 | 10% Solution | 6 hours | 5.56 | 5.63 | 6.19 | 6.13 | 5.08 | 5.18 | 5.82 | 0.46 |
| 1 | Infection control | 12 hours | 7.03 | 7.22 | 7.33 | 7.28 | 7.04 | 6.92 | 7.16 | 0.16 |
| 2 | Vehicle Control | 12 hours | 7.14 | 7.09 | 6.98 | 6.82 | 7.18 | 7.18 | 7.08 | 0.14 |
| 3 | 10% Gel | 12 hours | 6.13 | 5.93 | 5.49 | 5.00 | 5.21 | 5.04 | 5.68 | 0.47 |
| 4 | 10% Solution | 12 hours | 5.33 | 5.96 | 5.99 | 5.92 | 5.82 | 5.24 | 5.80 | 0.34 |
| 5 | 1% Soframycin | 12 hours | 6.01 | 5.85 | 6.71 | 6.46 | 6.93 | 6.81 | 6.61 | 0.44 |

**Table S10:** Detailed data from the multiple dose murine acute dermal infection with MRSA comparing 5% and 10% Nu-3 gels to infection and vehicle gel controls at 5 days and 10 days post infection.

|  |  |  | **Log_10_ CFU** | | | | | | | |
| --- | --- | --- | --- | --- | --- | --- | --- | --- | --- | --- |
| **Group** | **Treatment** | **Time** | **1** | **2** | **3** | **4** | **5** | **6** | **Mean** | **SD** |
| 1 | Infection control | 5 days | 6.83 | 8.16 | 7.13 | 8.20 | 7.10 | 6.94 | 7.76 | 0.62 |
| 2 | Vehicle BID | 5 days | 8.22 | 7.09 | 8.04 | 7.01 | 6.07 | 6.95 | 7.71 | 0.79 |
| 3 | 5% gel BID | 5 days | 4.97 | 4.64 | 4.82 | 4.54 | 4.68 | 4.59 | 4.73 | 0.16 |
| 4 | 10% gel bid | 5 days | 3.98 | 4.92 | 3.45 | 3.86 | 3.55 | 3.84 | 4.28 | 0.52 |
| 5 | Vehicle QD | 5 days | 8.47 | 6.96 | 6.44 | 7.90 | 6.35 | 7.04 | 7.82 | 0.83 |
| 6 | 5% Gel QD | 5 days | 6.17 | 5.90 | 6.01 | 4.77 | 4.31 | 4.72 | 5.76 | 0.80 |
| 7 | 10% Gel QD | 5 days | 4.69 | 5.41 | 5.31 | 5.37 | 4.69 | 4.49 | 5.14 | 0.41 |
| 1 | Vehicle BID | 10 days | 8.22 | 7.09 | 8.04 | 7.01 | 6.07 | 6.95 | 7.71 | 0.79 |
| 2 | 5% gel BID | 10 days | 5.04 | 5.68 | 6.50 | 6.83 | 5.64 | 5.63 | 6.28 | 0.66 |
| 3 | Vehicle QD | 10 days | 8.47 | 6.96 | 6.44 | 7.90 | 6.35 | 7.04 | 7.82 | 0.83 |
| 4 | 5% Gel QD | 10 days | 5.37 | 7.06 | 7.33 | 6.97 | 7.02 | 5.58 | 6.95 | 0.85 |
